# The effect of a dual or a triple antithrombotic therapy with apixaban on thrombus formation in vivo and in an ex vivo perfusion chamber model: An open-label, controlled, sequential study

**DOI:** 10.1097/MD.0000000000004145

**Published:** 2016-07-08

**Authors:** Stefan Weisshaar, Brigitte Litschauer, Sebastian Bucher, Martin Riesenhuber, Stylianos Kapiotis, Paul Alexander Kyrle, Michael Wolzt

**Affiliations:** a Department of Clinical Pharmacology; b Clinical Institute of Laboratory Medicine; c Department of Internal Medicine I, Division of Haematology, Medical University of Vienna, Austria.

**Keywords:** apixaban, aspirin, combination, drug therapy, ticagrelor

## Abstract

**Background:**

There is a need to optimize pharmacological treatment in patients with acute coronary syndrome and concomitant atrial fibrillation, in particular with newer antithrombotic medicines. We have therefore studied if dual or triple combination of antithrombotic agents exert similar effects on coagulation activation in an in vivo model in the skin microvasculature and in an ex vivo perfusion chamber.

**Methods and Results:**

Shed blood platelet activation (β-thromboglobulin [β-TG]), thrombin generation (thrombin-antithrombin complex [TAT]) and volume as well as markers of thrombus size (D-dimer) and its platelet content (P-selectin) in a perfusion chamber were studied in a sequential, open-label, parallel group trial in 40 healthy male volunteers (n = 20 per group). Subjects received ticagrelor and apixaban without or with acetylsalicylic acid (ASA). Outcome parameters were assessed at 3 hours after therapy dosing, and at steady-state trough and peak conditions.

A triple or dual therapy induced a comparable decrease in shed blood β-TG at 3 hours after therapy dosing but was more pronounced at steady-state conditions with the more intense treatment combination. During both antithrombotic regimens a similarly sustained inhibition in thrombin generation was observed which was accompanied by comparable increases in shed blood volume. In contrast, no treatment effect could be observed in the perfusion chamber experiment.

**Conclusion:**

Ticagrelor and apixaban with or without ASA inhibit platelet activation and thrombin formation in vivo in healthy subjects. Platelet inhibition was greater at steady-state conditions after triple therapy administration.

## 1 Introduction

Atrial fibrillation (AF) is the most frequent arrhythmia in cardiac patients. The vast majority of these subjects require long-term oral anticoagulation for prevention of thromboembolism with vitamin K antagonists (VKA) or nonvitamin K oral anticoagulants (NOAC) such as apixaban, a direct factor Xa (FXa) inhibitor.^[1–5]^ Apixaban has been shown to be superior to VKA regarding thromboembolic events and bleeding complications in patients with AF.^[6]^

Dual antiplatelet therapy (DAPT) with acetylsalicylic acid (ASA) and a P2Y_12_ receptor inhibitor such as clopidogrel, prasugrel, or ticagrelor has become a mainstay of the treatment in patients for secondary prevention after acute coronary syndrome (ACS) to lower the risk of stent thrombosis.^[7–10]^ Ticagrelor, a reversible P2Y_12_ antagonist, has demonstrated a superior risk/benefit profile compared to clopidogrel and is also considered to reduce the incidence of coronary stent thrombosis.^[11,12]^

AF patients who undergo percutaneous coronary intervention (PCI) require additional antiplatelet therapy. However, an optimal antithrombotic strategy for AF patients with PCI is not established. Retrospective data have shown that a triple therapy with DAPT + VKA is associated with an increased bleeding risk, but withholding oral anticoagulation increases thromboembolic events.^[13–18]^ Thus, recommendations from different guidelines are controversial. The European Society of Cardiology (ESC) suggests antithrombotic triple therapy for at least 1 month after PCI,^[19]^ whereas the American College of Cardiology Foundation/ American Heart Association (ACC/ AHA) is more reserved in recommending a regimen with VKA + ASA + clopidogrel.^[1]^ At present, the use of newer P2Y_12_ antagonists as part of triple therapy combinations lacks clinical evidence.^[19]^ The WOEST trial has shown that AF patients that underwent PCI and received a dual therapy (VKA + clopidogrel) without ASA may benefit from a less intense pharmacological treatment, which resulted in a lower bleeding risk accompanied by similar rates of thrombotic events compared to a triple therapy.^[16]^ Whether these positive effects can also be extrapolated to combinations of other anticoagulants is unknown.

Different experimental models such as the shed blood technique or the perfusion chamber method have been employed to investigate the (combined) effects of anticoagulants on biomarkers of platelet activation, thrombin generation, and thrombus size under physiologic conditions.^[20–22]^ The shed blood technique allows us to assess the hemostatic system activation in humans in vivo. This model utilizes standardized skin microvasculature incisions with collection of the emerging blood for assessment of platelet activation and thrombin formation.^[23–30]^

The perfusion chamber model, as originally described by Badimon,^[31]^ is an ex vivo model of thrombosis that enables us to study clot formation at low and high shear rates simulating flow conditions of the venous system and in stenosed arteries, respectively. Previous studies have shown that the size of a thrombus formed on a thrombogenic surface is directly correlated with D-dimer concentrations of the plasmin-digested clot.^[32,33]^ Measurement of P-selectin has been evaluated as a biomarker to indicate the platelet content of the thrombus formed in the perfusion chamber.^[34]^

This trial aimed to investigate the effect of orally administered ticagrelor and apixaban with or without ASA in healthy volunteers on in vivo hemostatic system inhibition in shed blood and on thrombus formation in an ex vivo perfusion chamber model at high and low shear rate.

## 2 Methods

Approval for the trial was provided by the Ethics Committee of the Medical University of Vienna, Austria (EK 1984/2013), and the national competent authority. The study protocol complies with the Declaration of Helsinki, including current revisions and the ICH Good Clinical Practice Guidelines. It is registered at ClinicalTrials.gov (NCT02080858) and at the European Clinical Trials database (EudraCT 2013-004613-40). All study participants gave written informed consent.

### 2.1 Study population

**Figure 1:**
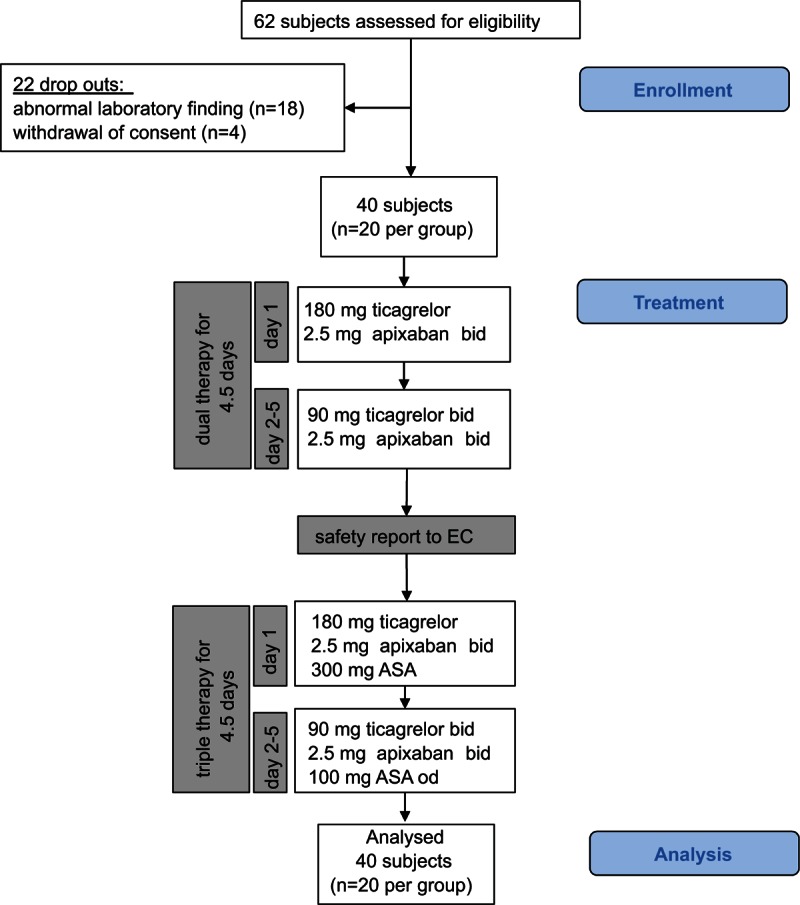
Study flowchart. ASA = acetylsalicylic acid, bid = twice daily, EC = Ethics Committee, od = once daily.

In total, 62 healthy, nonsmoking male volunteers aged 18 to 40 years with a body mass index (BMI) of 18 to 27 kg/m^2^ were evaluated for eligibility to assure that 40 subjects entered the active treatment phase (Fig. 1). Participants were excluded if they had any significant laboratory or physical finding; a history of renal, hepatic, gastrointestinal or cardiac impairment; disorders with an increased risk of bleeding or coagulation diseases; use of any medication within 2 weeks prior inclusion. A screening visit was scheduled 7 to 21 days before the first study. Subjects abstained from alcohol throughout the study.

### 2.2 Study design and study drugs

Between March 2014 and October 2014 a single center, prospective, controlled, analyst-blinded study in a sequential fashion (study A: dual therapy; study B: triple therapy) was conducted at the Department of Clinical Pharmacology, Medical University of Vienna, Austria. Subjects received ticagrelor (Brilique^®^, Astra Zeneca AB, Södertälje, Sweden) and apixaban (Eliquis^®^, Bristol-Myers Squibb/ Pfizer, Uxbridge, United Kingdom) without (study A; n = 20) or with ASA (Thrombo ASS^®^, Lannacher Heilmittel GmbH, Lannach, Austria) (study B; n = 20) for 4 days to allow steady-state conditions on the fifth study day. A sequential study design was implemented as the Ethics Committee was concerned with safety aspects and required a detailed adverse event (AE) interim report before commencement of the triple therapy cohort (Fig. 1). Subjects were consecutively allocated to the study cohorts according to their screening number.

On study day 1, subjects received a loading dose of 180 mg ticagrelor in combination with apixaban 2.5 mg twice daily (bid) and a 300 mg loading dose ASA (only study B). From day 2 until day 4 ticagrelor 90 mg bid, apixaban 2.5 mg bid without (A) or with 100 mg ASA (B) was administered. On day 5 subjects received the medicines under investigation once (ASA only in study B) to assess steady-state trough (pre-dose) and expected peak conditions at 3 h postdosing. An end-of-study (EOS) visit was scheduled earliest 4 days after study drug discontinuation. The dose for apixaban was chosen according to the recommendations of the ESC guidelines^[19]^ suggesting a lower dose intensity of NOAC in triple therapy combinations. This is also consistent with ongoing trials investigating pharmacological regimens with ticagrelor + rivaroxaban.^[35]^

### 2.3 Study endpoints

The primary objective included comparisons of changes in the main outcome parameter, shed blood β-thromboglobulin (β-TG), from baseline (immediately before loading dose) between time points during antithrombotic therapy and treatment groups. β-TG, a protein released during platelet activation from α-granules, was chosen as well-established shed blood platelet marker to ensure that different modes of action of the medicines under study are picked up. It has been shown previously that β-TG can detect pharmacodynamic effects of ASA, ticagrelor, and NOAC.^[20,23,36]^ In addition, a major effect of antiplatelet drugs on shed blood thrombin generation was not expected.^[20]^

Secondary objectives were to compare concentrations of thrombin–antithrombin complex (TAT) in shed blood as well as D-dimer and P-selectin levels of the plasmin dissolved thrombi from the perfusion chamber. TAT is created by cleavage of a scissile bond from antithrombin III by thrombin and has been shown to be more sensitive to detect difference in thrombin generation than other shed blood parameters such as prothrombin fragment 1+2.^[20,37]^ D-dimer and P-selectin are markers of thrombus size and thrombus platelet deposition, respectively.^[32–34]^

Other secondary outcome parameters included shed blood volume, pharmacokinetics of apixaban, prothrombin time (PT), activated partial thromboplastin time (aPTT), and safety.

### 2.4 Pharmacodynamics and Pharmacokinetics

Assessment of parameters for pharmacodynamics and kinetics was performed at baseline, 3 h after first drug administration on day 1, at steady state on study day 5 and at expected peak steady-state conditions at 3 h after dosing on day 5.

### 2.5 Shed blood

Shed blood collections were performed as described in our previous trial^[20]^ based on a technique by Mielke et al^[38]^ Briefly, a sphygmanometer cuff was placed at the upper arm and inflated to 45 mm Hg. Two incisions, 5 mm long and 1 mm deep, were placed on the lateral aspect of the forearm parallel to the antecubital crease by using disposable standard bleeding time devices (Surgicutt^®^; ITC, Edison, NJ). In order to avoid activation of platelets and coagulation factors due to skin contact, shed blood was collected directly from the edge of the skin at 15 seconds intervals over a period of 4 minutes using plastic pipette tips (Greiner Bio One International AG, Kremsmünster, Austria). Four minutes of sampling was chosen because it has been demonstrated that shed blood markers reach plateau levels within this period.^[39]^ The blood from each incision site was transferred immediately into 2 separate ice-cooled plastic tubes containing 100 μL stop solution (3.8% sodiumcitrate, 0.5% indomethacin dissolved with ethanol, 10% aprotinin [400 μM, 10000 KIU/mL]; all from Sigma Aldrich, MO) to prevent further thrombin generation or platelet activation in the tubes. Bleeding time incision experiments were performed by the same investigator to reduce operator variability. Shed blood volume per incision was estimated from pre- and postsampling tube weights and total shed blood volume was calculated**.** The test tubes were immediately centrifuged at 14,000 g for 10 minutes at 4 °C. The plasma was separated, pooled, and stored at –80 °C until analysis. β-TG and TAT plasma concentrations in shed blood were determined by adjustment of ELISA readings with a correction factor calculated from the ratio of stop solution versus individual shed blood amount in the collection tubes: correction factor = (stop solution in both tubes [200 μL]/ total shed blood volume [μL])+1.

### 2.6 Perfusion chamber experiments at low and high shear rate

An ex vivo perfusion chamber model as described by Badimon et al^[31]^ was used to investigate the effect of the medicines under study on thrombus size and its platelet content. For each experiment (low or high shear rate), 2 serially placed chambers with a longitudinal cylindrical hole to mimic rheological conditions for venous blood flow with an estimated shear rate of 212 s^−1^ or for moderately stenotic arterial flow (1690 s^−1^) were heated up to 37 °C. Each chamber contained a piece of a de-endothelialized pig aorta tunica media as the thrombogenic surface. Before the blood perfusions, the chambers were flushed with 0.9% sodium chloride to ensure that there were neither leaks nor air bubbles in the system. Subsequently, blood was drawn directly from the study participants from a cubital vein through an 18G cannula with a peristaltic pump (Masterflex^®^ L/S™, Cole-Parmer Instrument Company, IL) placed distal from the chambers. The blood was perfused over the denuded pig aorta for 5 minutes at 10 mL/min, followed by a 30 second perfusion with 0.9% sodium chloride to remove unbonded thrombotic particles.

Thrombus size on the tunica media was assessed by measurement of the D-dimer concentrations of the plasmin dissolved thrombi. D-dimer levels have been shown to directly correlate with the amount of fibrin deposited and thus the size of the thrombus formed in the perfusion chamber.^[33,34]^ The clot formed on the pig aorta was degraded with plasmin (500 μL of 0.1 M Tris-buffered saline [pH 7.4, Merck KGaA, Darmstadt, Germany] and 50 μL plasmin [10 U/mL, Hyphen Biomed, Neuville-sur-Oise, France]). The tubes were placed in a shaking water bath (37 °C) for 45 minutes and the protease activity was stopped afterwards by addition of 50 μL of aprotinin (400 μM, Sigma Aldrich, MO). The mixture was centrifuged at 10,000 g for 5 minutes at 4 °C and the supernatant was stored at –80 °C until analysis.

The remaining cell pellet was further processed as described previously by Bossavy et al^[34]^ with minor adaptations for assessment of P-selectin concentrations. It has been shown that there is a direct correlation between thrombus platelet content and measured P-selectin levels from the lysed thrombocytes. The cell pellet was dissolved in 500 μL of a lytic buffer (PBS [Life Technologies, Carlsbad, CA] with 1% Triton X-100 [Sigma Aldrich, MO], 1 mM EDTA [Sigma Aldrich, MO], 0.05% sodium azide [Sigma Aldrich, MO], Complete^®^ protease inhibitor cocktail tablets [Roche Diagnostics, Risch, Switzerland]) 3 times frozen in liquid nitrogen and then sonicated at 4 °C at 35 KHz for 4 minutes. Subsequently, the tubes were centrifuged at 10,000 g for 5 minutes at 4 °C and the supernatant was stored at –80 °C until assayed for P-selectin determination.

### 2.7 Venous blood

Blood for estimated apixaban plasma concentrations measured as anti-Xa activity, aPTT, and PT was collected without tourniquet from the 18G cannula in 3.8% sodium citrate tubes (Greiner Bio One International AG, Kremsmünster, Austria) immediately before the perfusion chamber experiments were carried out.

Venous blood samples were centrifuged at 1500 g for 15 min at room temperature and the separated plasma was stored at –80 °C until batch analysis.

### 2.8 Analytical methods and assays

Screening and EOS laboratory analysis were performed at the Clinical Institute of Laboratory Medicine, General Hospital Vienna, Austria, according to standard operating procedures. All other parameters were analyzed by Labcon GmbH (Vienna, Austria) using validated assays: ASSERACHROM^®^ β-TG test kit (Diagnostica Stago, Asniéres, France) for β-TG assessment, TAT was assessed using the Enzygnost^®^ TAT micro kit (Siemens GmbH, Munich, Germany), D-dimer was analyzed with the TECHNOZYM^®^ D-dimer ELISA (Technoclone, Vienna, Austria) and P-selectin was determined using the Human sP-selectin Platinum ELISA (eBioscience, CA). The detections ranges were 117.5 to 1880 U/mL for β-TG, 20 to 600 ng/mL for TAT, 189.7 to 1365 ng/mL for D-dimer, and 0.63 to 40 ng/mL for P-selectin.

Apixaban pharmacokinetics were assessed as anti-Xa activity with an ACL TOP500 coagulation analyser (Instrumentation Laboratory, Vienna, Austria) using apixaban as assay standard with a lower limit of quantification (LLQ) <10 ng/mL.

The ACL TOP500 coagulation analyzer was also used for aPTT and PT measurements using HemosIL^®^ SynthASil (Instrumentation Laboratory, Vienna, Austria; [aPTT]) and HemosIL^®^RecombiPlasTin 2G (Instrumentation Laboratory, Vienna, Austria; [PT]) as reagents. The normal reference range for venous aPTT was 25.1 to 36.5 s and 70 to 130 % for venous PT.

### 2.9 Sample size and statistical analysis

Sample size calculation was based on the primary outcome parameter shed blood β-TG using study population data from our previous trial.^[20]^ Assuming a within-group standard deviation (SD) of 21%, a sample size of 20 subjects per group will have 80% power to detect a mean difference of 20% between dual or triple antithrombotic therapy with a 2-sample *t* test at a significance level of 5% (2-tailed). The calculated sample size of 20 included drop-out of 2 subjects.

Statistical evaluations were performed with SPSS 22 for Macintosh (IBM Cooperation, New York, NY). Data sets were analyzed descriptively and are presented as mean and SD. Normal distributions were examined with the Kolmogorov–Smirnov test. Comparisons of screening laboratory data and demographic parameters were performed using unpaired *t* tests. Shed blood parameters (β-TG, TAT, volume) and perfusion chamber markers (D-dimer, P-selectin) were analyzed by a repeated measures analysis of variance (ANOVA) with adjustments for multiple comparisons using the Bonferroni procedure. Time point was considered as within-subject factor, treatment as the between-group factor and pre-dose (baseline) levels were used as covariate in the ANOVA model. Significant interactions between group and time point were further analyzed by post hoc comparisons applying unpaired *t* tests. An adjusted 2-sided *P* value < 0.05 was considered to be statistically significant.

## 3 Results

### 3.1 Study population

Demographic data and screening laboratory characteristics of the 40 study participants that completed the study are presented in Table 1. Parameters did not differ between treatment groups (*P* > 0.05 for all parameters, unpaired *t* test).

**Table 1:**
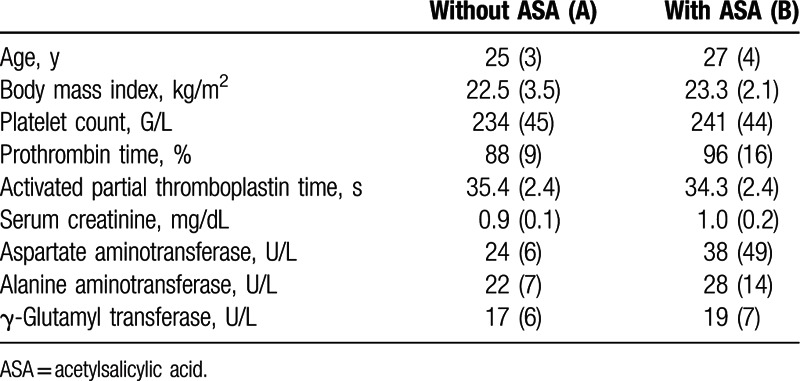
Demographic data and laboratory characteristics of study participants. Data are presented as mean and standard deviation (n = 20 per group).

### 3.2 Effects on shed blood markers

Shed blood data (β-TG, TAT, volume) are summarized in Table 2. Baseline (pre-dose) concentrations of all shed blood outcome parameters were comparable between study cohorts (*P* > 0.05, ANOVA).

**Table 2:**
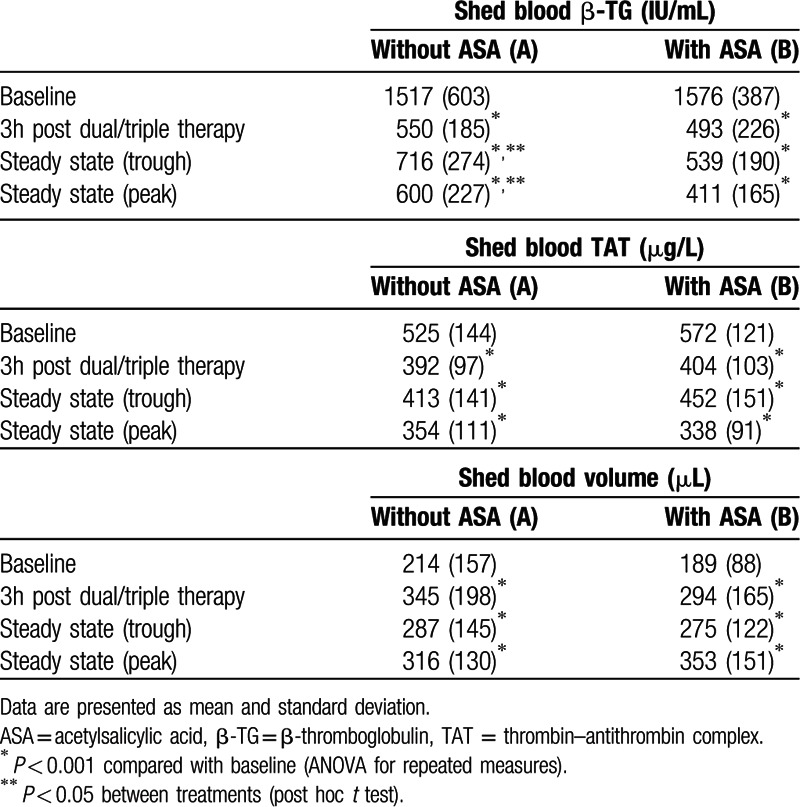
Shed blood β-thromboglobulin (β-TG) concentrations, thrombin–antithrombin complex (TAT) concentrations and total shed blood volume (n = 20 per group).

### 3.3 Shed blood β-TG

**Figure 2:**
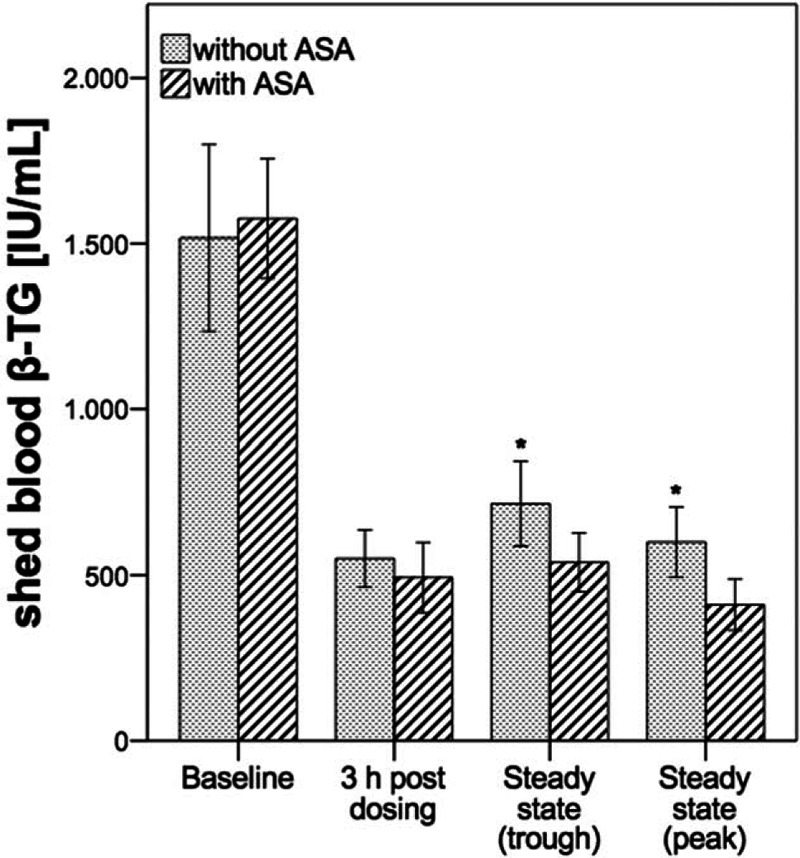
Shed blood β-thromboglobulin (β-TG) concentrations by the treatment group and time point. Data are presented as mean and 95% confidence interval. ^∗^*P* < 0.05 between treatments (post hoc *t* test). (n = 20 per group). β-TG = β-thromboglobulin.

β-TG levels at different time points are shown in Fig. 2. Administration of a dual therapy without ASA (A) or a triple antithrombotic therapy with ASA (B) significantly decreased β-TG release at all time points compared with predose concentrations (*P* < 0.001, ANOVA; Table 2) with significant differences between groups (*P* = 0.011, ANOVA). At 3 hours after a loading dose of ticagrelor in combination with apixaban (A) β-TG was reduced by 64% and with an additional ASA loading dose (B) by 69% indicating a comparable change between study groups (*P* = 0.07 A vs B, post hoc *t* test). At trough steady-state mean β-TG concentrations remained below individual baseline levels (53% [A] vs 66% [B]) with less pronounced β-TG suppression in the dual therapy group versus triple antithrombotic treatment (*P* = 0.035, post hoc *t* test). This pattern was also observed at steady-state peak conditions with a mean decrease in β-TG concentrations of 60% (A) and 74% (B), respectively (*P* = 0.013 A vs B, post hoc *t* test).

### 3.4 Shed blood TAT

**Figure 3:**
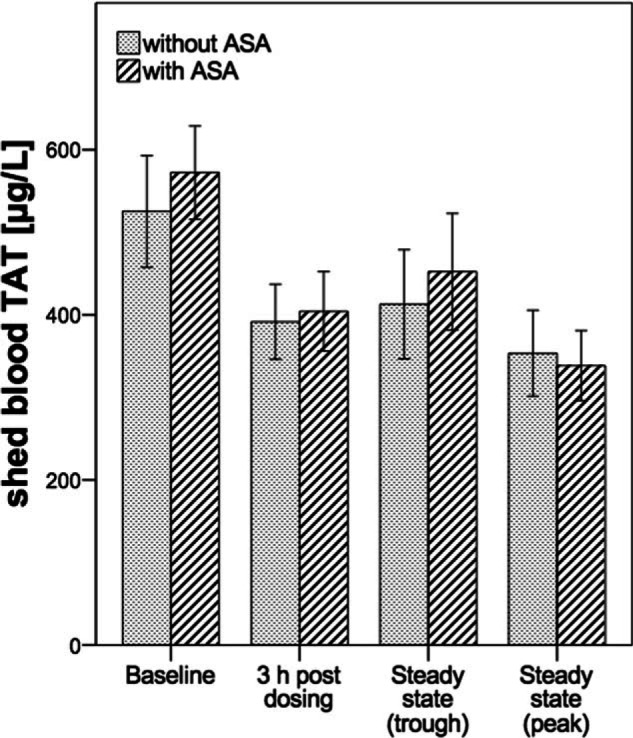
Shed blood thrombin–antithrombin complex (TAT) concentrations by the treatment group and time point. Data are presented as mean and 95% confidence interval. (n = 20 per group). TAT = thrombin–antithrombin complex.

Data are presented in Fig. 3. Similar to β-TG, dual or triple therapy reduced TAT levels as compared with individual baseline conditions at all time points (*P* < 0.001, ANOVA; Table 2). Following a ticagrelor loading dose combined with apixaban (A) mean TAT was 25% below baseline at 3 hours after administration. At 3 hours after triple therapy dosing (B) the observed reduction in TAT levels was similar (–29%). At steady-state trough TAT concentrations remained significantly reduced by 21% below baseline in both study cohorts. At peak steady state the maximum decrease in TAT with reductions of 33% (A) and 41% (B) below predose were noted. No differences across treatment groups could be detected at any time point during antithrombotic therapy (*P* = 0.82 for group differences; ANOVA).

### 3.5 Shed blood volume

Increases in shed blood volume were noted at all time points during antithrombotic therapy as compared with predose values (*P* < 0.001, ANOVA) ranging from +34–61% (A) to +46–87% (B). Shed blood volume increases were smallest at trough steady state (+34% [A] vs +46% [B]) whereas time points of maximum increases varied across cohorts and were at 3 h after therapy initiation (A) and at steady-state peak conditions (B). There were no differences between dual or triple therapy at corresponding time points (*P* = 0.66 for group differences, ANOVA).

### 3.6 Fibrin deposition and thrombus platelet content from the perfusion chamber

Data from the perfusion chamber experiments at high and low shear rate are presented in Table 3. Administration of a dual or triple antithrombotic therapy did not affect thrombus D-dimer concentrations or P-selectin levels as a marker of thrombus platelet content as compared to baseline conditions (*P* > 0.05, ANOVA). The observed discrepancies between different shear rates and treatments with inconclusive changes in these markers might be due to the high inter-subject variability of the measurements. As a result, this may have masked the true effect of the medicines under study and might also explain that no group differences could have been detected except for P-selectin concentrations at a low shear rate (*P* = 0.002, ANOVA) at 3 h after antithrombotic therapy initiation (*P* = 0.018, post hoc *t* test). However, this should be interpreted cautiously, as these changes are paralleled by greater differences between study cohorts with an increase during dual therapy (+7% vs. baseline) and a decrease (–19%) in the triple therapy group.

**Table 3:**
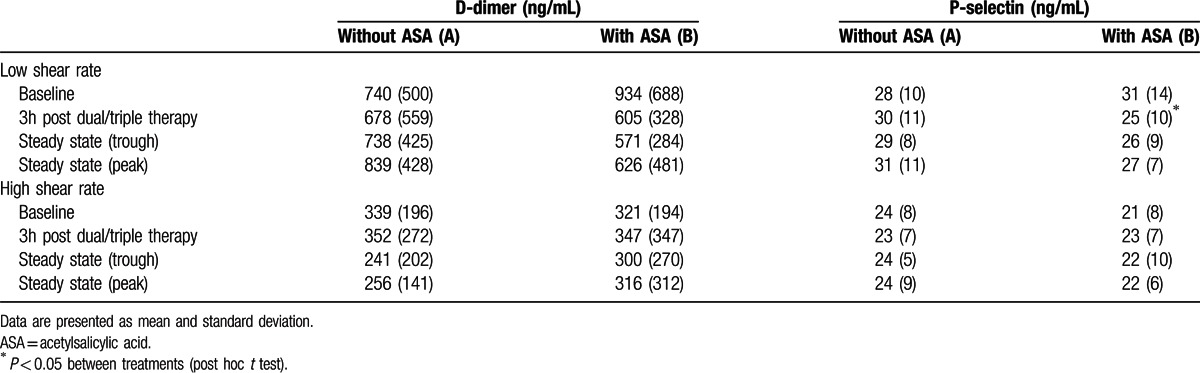
Thrombus D-dimer concentrations and P-selectin concentrations at low and high shear rates in the perfusion chamber after treatment with ticagrelor and apixaban without (A) or with (B) acetylsalicylic acid (ASA) (n = 20 per group).

### 3.7 Apixaban pharmacokinetics and pharmacodynamic parameters from venous blood

Apixaban plasma concentrations, as estimated by anti-Xa activity with apixaban as a calibrator, PT and aPTT at corresponding time points and groups are summarized in Table 4. No group differences could be detected in apixaban levels (*P* = 0.63, ANOVA). For PT and aPTT no statistical analysis was performed and these markers are only presented descriptively. Pharmacodynamic parameters responded in a concentration-dependent manner with maximum changes at peak apixaban levels.

**Table 4:**
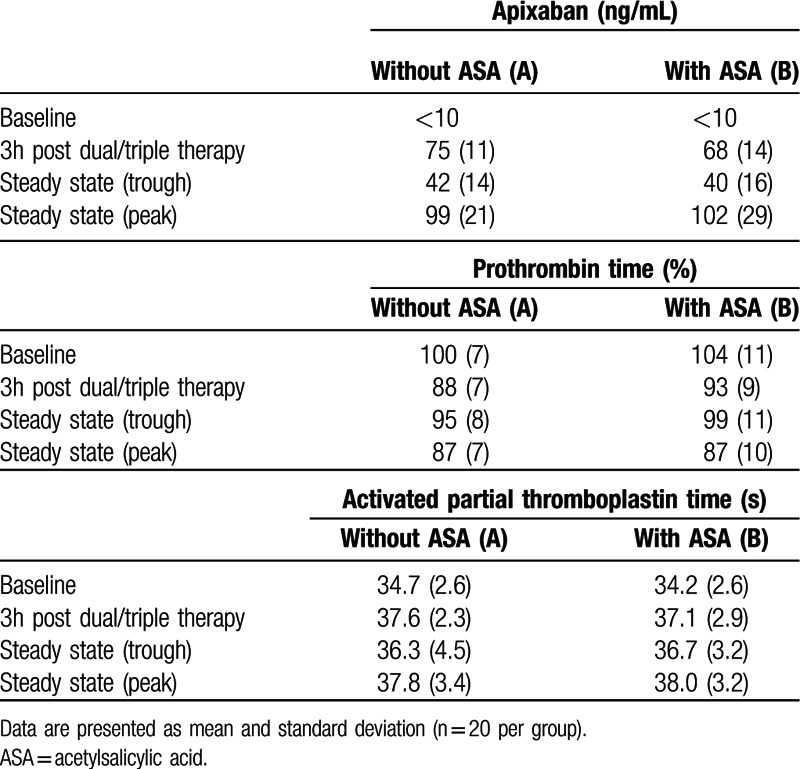
Apixaban plasma concentrations (estimated as anti-Xa activity with apixaban as a calibrator) and pharmacodynamics (prothrombin time, activated partial thromboplastin time) during antithrombotic dual (A) or triple therapy (B).

### 3.8 Safety

The incidence of AEs was low in both study cohorts and the medicines under investigation were well tolerated. Six subjects (15%) reported a total of 10 AEs (group A n = 3; B = 7) that were mild in severity and resolved spontaneously. Three AEs were considered to be possibly related to the study drugs (hematoma, prolonged bleeding from shed blood incision site, dyspnea) and occurred only during antithrombotic triple therapy.

## 4 Discussion

In this controlled study, orally administered ticagrelor and apixaban with or without ASA induced in vivo an effective and prolonged attenuation of platelet activation (β-TG) and thrombin generation (TAT) in shed blood in healthy volunteers. Inhibition of β-TG release was similar after an acute dose during both dual and triple antithrombotic therapy but more pronounced at steady-state trough and peak conditions with the more intense combination treatment (ticagrelor + apixaban + ASA). Duration of antithrombotic therapy had no additional impact on TAT formation. In the ex vivo perfusion chamber, no effect on thrombus size (D-dimer) and its platelet content (P-selectin) could be detected.

We studied the impact of combined antithrombotic therapy by 2 different experimental approaches that have been used previously to determine pharmacodynamic effects of antithrombotic agents at the site of plug formation: in vivo, using the shed blood technique^[20,23–25,28,29]^ and ex vivo, applying a perfusion chamber model.^[21,22,40]^ Shed blood allows for assessment of hemostatic system activation in the microcirculation by local skin injury following tissue factor-induced blood coagulation.^[26]^ This approach has been shown to be more sensitive to distinguish pharmacodynamic differences than in vitro activation of venous blood.^[24,30]^ In contrast, the ex vivo perfusion chamber enables assessment of clots formed under physiologic flow conditions mimicking venous and arterial vessels by measurement of markers of thrombin generation and platelet disposition of the plasmin-digested thrombus.^[34]^

Our shed blood data suggest a similar efficiency of an acute ticagrelor loading dose + apixaban with or without ASA on platelet inhibition, whereas at steady-state conditions a triple therapy induces a stronger attenuation of β-TG release as compared with dual therapy. We have shown previously that ticagrelor and NOAC contribute additively and concentration dependent to platelet inhibition^[20]^ indicating that ticagrelor loading combined with apixaban might have concealed the acute impact of ASA. This might be due to the potent effect of these novel antithrombotic medicines on inhibition of β-TG release resulting in similar changes between dual and triple anticoagulation. However, the magnitude of ticagrelor or apixaban on shed blood pharmacodynamics may differ at assessed time points due to different pharmacokinetics of the medicines under study.^[41,42]^

Changes in shed blood β-TG release at steady state with an apixaban dose of 2.5 mg bid in triple therapy combination are consistent with other antithrombotic triple therapy regimens of ticagrelor + ASA + (N)OAC at approved doses for AF in a comparable study cohort.^[20]^ Thus, it cannot be ruled out that administration of a higher apixaban dose may abrogate differences between triple and dual therapy at steady-state conditions due to a more potent apixaban-induced attenuation of β-TG. Although apixaban has not been tested so far using the shed blood technique, the apixaban dosing scheme in this study may also explain the inconsistency with previous data regarding the greater β-TG inhibition during an acute situation at 3 hours after therapy initiation with high doses of other NOAC such as dabigatran or rivaroxaban.^[20]^ However, similar apixaban concentrations between study cohorts in this trial suggest that the differences in β-TG at steady state are not affected by NOAC. As variations in ticagrelor plasma concentrations have been previously shown to be of little if any influence on shed blood markers in healthy male subjects during combined antithrombotic therapy,^[20]^ we did not assess ticagrelor pharmacokinetics in this study. In conclusion, the differences in shed blood β-TG observed at steady state may be attributed to the additive effect of ASA in study cohort B.

As expected, TAT formation, that has been shown to be unaffected by platelet inhibitors in our previous trial, was similar across study cohorts at all assessed time points. The concentration-dependent inhibitory effect of apixaban on TAT formation is in agreement with other NOAC that showed a greater inhibition at higher doses.^[20,24,30]^ The discrepancy between venous pharmacodynamics, that were within (PT) or slightly above the normal range (aPTT), after NOAC administration and the sustained inhibition in shed blood thrombin generation may be explained by apixaban’′s mechanism of action and the different activation states of the coagulation system. Subendothelial in vivo tissue factor exposure results in an immediate activation of the clotting system at the site of plug formation with rapid and amplified thrombin generation.^[26]^ It is likely that apixaban, that targets free and prothrombinase complex bound FXa, more effectively attenuates pharmacodynamic markers at activated coagulation conditions (shed blood) than in a resting state (venous blood) where the amplification mechanism is less developed, smaller amounts of thrombin are generated, and apixaban's potential of direct FXa inhibition is not fully exploited accordingly.^[24,43–45]^ This is consistent with previous observations suggesting that traditional clotting assays may not be adequately sensitive for evaluation of the pharmacodynamic effects of apixaban.^[46,47]^

Similar to TAT formation, effects on shed blood volume data were not different between triple or dual antithrombotic therapy. This might indicate a comparable bleeding potential independent from ASA administration and would be in agreement with available data from a prospective trial in a population at risk.^[16]^ However, shed blood volume is subject to substantial between-subject variability and the study was not powered to detect group differences in this secondary outcome parameter. Furthermore, it is likely that conditions of plasmatic coagulation and platelet activation in the skin microvasculature differ from that seen in stenosed arteries due to higher shear rates in the arterial vascular bed. Thus, it remains unclear whether the observed similar effects on bleeding volumes across study cohorts represent a comparable safety profile of the treatment regimens under study as shed blood markers can only act as surrogates. Consequently, clinical evidence from trials evaluating these novel antithrombotic medicines in the subset of AF + ACS patients is required to confirm our findings.

In contrast to the shed blood model, we could not detect any treatment effect on thrombus size or thrombus platelet content in the ex vivo perfusion chamber at both low and high shear rates after administration of a dual or a triple therapy. This is at variance with other antithrombotic drugs such as enoxaparin, rivaroxaban, or clopidogrel^[22,40]^ and may have resulted from the high variability across study cohorts in the assessed outcome parameters and the low dose of apixaban administered. It cannot be ruled out that using a deendothelized pig aorta with irregularities at different degrees on the surfaces of the manually prepared strips have influenced clot formation during blood perfusion.

We conducted this trial in a healthy population rather than cardiac patients requiring antithrombotic triple therapy due to ethical concerns in discontinuing a pre-existing anticoagulation. Thus, it cannot be excluded that results in patients might differ due to impaired hepatic or renal function and the inter- and intra-subject variability in patients with AF and ACS is probably larger than in this healthy cohort under study.

Our results suggest that in an acute situation a loading dose of ticagrelor in combination with low-dose apixaban may be sufficient for rapid and sustained inhibition of both platelet and coagulation activation in patients. The shed blood model may provide insight into effective combinations of antithrombotic therapies to prevent thromboembolic events.

### 4.1 Limitations

The trial was conducted in healthy volunteers. Thus, it remains unclear if our results can be extrapolated to a population at risk with concomitant medication and comorbidities that may affect the metabolism of the study drugs and their pharmacodynamic response. Additionally, short-term administration of a triple or a dual therapy did not induce major bleedings and the shed blood volume response was similar. We cannot conclude that these drug combinations have similar safety profiles as they have not been assessed during long-term treatment and it has not yet been confirmed that shed blood volume as a surrogate of bleeding correlates with clinical bleeds.

## 5 Conclusion

A triple or dual therapy of ticagrelor and apixaban with or without ASA effectively inhibits platelet activation and thrombin generation at the site of plug formation in healthy subjects. Greater platelet inhibition was detected at steady-state conditions after administration of triple therapy. It remains unclear whether or not these differences affect the risk for clinical bleeds and further trials are required to confirm the finding of shed blood volume.
